# Mechanisms of wound closure following acute arm injury in *Octopus vulgaris*

**DOI:** 10.1186/s40851-016-0044-5

**Published:** 2016-03-29

**Authors:** Tanya J. Shaw, Molly Osborne, Giovanna Ponte, Graziano Fiorito, Paul L.R. Andrews

**Affiliations:** Division of Biomedical Sciences, St George’s University of London, Cranmer Terrace, London, SW17 0RE UK; Stazione Zoologica Anton Dohrn, Villa Comunale, 80121 Naples, Italy; King’s College London-Centre for Molecular and Cellular Biology of Inflammation, New Hunt’s House, Great Maze Pond, London, SE1 1UL UK

**Keywords:** Arm, Cell death, Cephalopod, Healing, Invertebrate, Microscopy, *Octopus vulgaris*, Regeneration, Wound repair

## Abstract

**Background:**

Octopoda utilise their arms for a diverse range of functions, including locomotion, hunting, defence, exploration, reproduction, and grooming. However the natural environment contains numerous threats to the integrity of arms, including predators and prey during capture. Impressively, octopoda are able to close open wounds in an aquatic environment and can fully regenerate arms. The regrowth phase of cephalopod arm regeneration has been grossly described; however, there is little information about the acute local response that occurs following an amputation injury comparable to that which frequently occurs in the wild.

**Methods:**

Adult *Octopus vulgaris* caught in the Bay of Naples were anaesthetised, the distal 10 % of an arm was surgically amputated, and wounded tissue was harvested from animals sacrificed at 2, 6, and 24 h post-amputation. The extent of wound closure was quantified, and the cell and tissue dynamics were observed histologically, by electron microscopy, as well as using ultrasound.

**Results:**

Macroscopic, ultrasonic and ultrastructural analyses showed extensive and significant contraction of the wound margins from the earliest time-point, evidenced by tissue puckering. By 6 h post amputation, the wound was 64.0 ± 17.2 % closed compared to 0 h wound area. Wound edge epithelial cells were also seen to be migrating over the wound bed, thus contributing to tissue repair. Temporary protection of the exposed tip in the form of a cellular, non-mucus plug was observed, and cell death was apparent within two hours of injury. At earlier time-points this was apparent in the skin and deeper muscle layers, but ultimately extended to the nerve cord by 24 h.

**Conclusions:**

This work has revealed that *O. vulgaris* ecologically relevant amputation wounds are rapidly repaired via numerous mechanisms that are evolutionarily conserved. The findings provide insights into the early processes of repair preparatory to regeneration. The presence of epithelial, chromatophore, vascular, muscle and neural tissue in the arms makes this a particularly interesting system in which to study acute responses to injury and subsequent regeneration.

**Electronic supplementary material:**

The online version of this article (doi:10.1186/s40851-016-0044-5) contains supplementary material, which is available to authorized users.

## Background

The limbs (arms and tentacles) are one of the most striking external features of the approximately 700 known extant species of cephalopods ranging from the numerous tentacles of nautiloids (e.g. *Nautilus pompilius)* to the eight arms and two tentacles of cuttlefish (e.g. *Sepia officinalis*) and squid (e.g. *Loligo pealii*) and the eight arms of octopoda (e.g. *Octopus vulgaris*, [1]). Due to the sea floor habitat of most species of octopus and the extensive use made of the arms for exploration, predation, and defence, the distal parts of the arms are the part of the body most likely to encounter a noxious challenge, and hence to be physically damaged. Octopuses are also known to remove arms by autophagy if they become trapped or damaged [2], and some species (*Abdopus aculeatus*, *Ameloctopus litoralis*) are able to autotomize their arms if traumatised [1, 3–5]. Surveys of octopuses caught in the wild show that arm damage is common. For example, in *O. vulgaris* 50 % of the animals caught in the Bay of Naples had some damage to one or more arms [6] and in the Pacific pygmy octopus, *Octopus digueti*, 26 % of animals had arm damage [7]. The high probability of damage to an appendage in the wild is one of the key evolutionary pressures for regeneration [8]. Although limb regeneration is a well-established phenomenon in members of all four orders of coleoid cephalopod (e.g. *S. officinalis* [9], *Sepioteuthis lessoniana* [10]; squid, *Omnastrephes bartrami* [11], *Octopoteuthis deletron* [12]; *O. vulgaris* [13]; and deep sea octopus, *Vampyroteuthis infernalis* [14]), it is rarely mentioned in reviews of comparative aspects of regeneration, despite the broad acknowledgment that knowledge of the underlying evolutionary processes would better inform our understanding of the fundamental mechanisms of regeneration [8, 15].

The arms in octopus function as “muscular hydrostats” [16] capable of marked changes in length under neural control. In *O. vulgaris* about 60 % of the neurones in the nervous system are located in the arms [17], with a centrally located axonal nerve cord comprising two adjacent axonal tracts containing motor and sensory nerves, and a more ventrally located chain of ganglia clustered in the region of each sucker to which they are connected [18]. In contrast to most other models of external appendage regeneration (e.g. axolotl forelimb, zebrafish fin), the arm in octopus provides a model without any skeletal reference point, and arguably a more complex behavioural repertoire for investigation of the relationship between physical and functional recovery.

To date, there have only been a few macroscopic and histological descriptions of the regenerative process of the octopus arm [13, 19] and healing of the mantle [20] following surgical injury. The redevelopment of appendages in other cephalopods [9, 21, 22] has also been described, and it is anticipated that many features of the tissue dynamics underpinning regeneration in these species will be shared. Despite detailed descriptions of appendage regrowth, there is a scarcity of information about the acute wound response to tissue damage (e.g. the first 24 h after injury), although it is known from other animal models that the acute response “sets the scene” for subsequent regeneration [23, 24]. In 1920, Lange provided a careful and eloquent description of these early tissue repair events in *O. vulgaris*; however, our current knowledge regarding the cellular basis of regeneration, together with improved microscopy and image acquisition capabilities, equip us to revisit this important topic. The inclusion of cephalopods in Directive 2010/63/EU regulating the use of animals for scientific purposes [25] also motivates an improved understanding of octopus wound repair, since this information will be informative in establishing guidelines for best surgical practice and post-operative care when cephalopods are used as research models [26]. The present study characterises wound closure over the first 24 h after removal of the distal 10 % of an arm in *O. vulgaris*. As with some vertebrate wounds, tissue contraction is confirmed as the key mechanism by which rapid closure is achieved; yet active cell migration by the epithelial cells at the wound edge was also initiated within 6 h of injury. A cellular plug overlaid the exposed arm tip, which is predicted to provide protection in a manner functionally analogous to a blood clot in vertebrates, as well as cells and extra-cellular matrix scaffolding for subsequent regenerative events.

## Methods

### Animals

*Octopus vulgaris*, Cuvier 1797 of both sexes caught in the Bay of Naples, Italy were used in this study. A total of 19 animals, 14 males and five females, were used in the main study, with a body weight range from 158 to 449 g (Gaussian distribution; mean ± standard error (SEM), 320 ± 20 g). Experimental groups were balanced for sex to the extent possible, and there was no significant difference in body weight between the groups at the three experimental time points (Kruskal-Wallis test, data not shown). As age determination is not possible in live cephalopods [27], it was not possible to account for this in the design. Studies were performed in September and February when the water temperature ranged between 17–21 °C. One male animal (body weight 570 g) was studied in June for in vivo high-resolution ultrasound imaging (details below). Animals were housed individually in tanks (30 × 50 × 100 cm) containing a den, constantly supplied with fresh sea water [28], and were fed with a small live crab (*Carcinus mediterraneus*) three times per week.

At the time the studies were performed, no national legislation was in place in Italy for regulation of research involving cephalopods. The care and welfare assessment of animals was consistent with best practice at the time the study was undertaken [29, 30] and took into account the then impending changes in the European Union legislation bringing cephalopods within the scope of regulations covering research involving living animals (Directive 2010/EU/63, [25, 26, 31]). Experimental design and animal care was discussed with the institution veterinarian who also monitored the study.

### Anaesthesia, surgery and humane killing

Immediately prior to surgery and to humane killing, the latency to attack a crab placed in the tank was measured to provide an indication of welfare following anaesthesia and surgery [32]. For surgery, animals were removed from their home tank by fine mesh net between 10.00 h and 12.00 h and anaesthetised in 5 L of 3.5 % magnesium chloride hexahydrate (Sigma-Aldrich) in sea water [33, 34]. Surgical anaesthesia occurred after 15–20 mins and was defined by an absence of a response to a noxious stimulus (mechanical), skin pallor (indicative of reduced central drive to chromatophores), muscle flaccidity, loss of righting reflex and suppression/absence of ventilation [29]. Animals were then placed in a shallow dish containing gassed 3.5 % magnesium chloride, arm L2 (second arm on the left side) was identified, the length measured (beak to tip; L2 mean arm length (±SEM): 405 ± 14 mm; *n* = 16, normal distribution verified). L2 lengths for two animals were not recorded, and in another animal R2 was used due to existing damage to L2; inclusion of this measurement results in a mean length of 404 ± 14 mm, *n* = 17. The distal 10 % was removed from the extended arm by transection using a new alcohol-sterilised Gillette Super Silver blade with the cut positioned to cut as vertically as possible to the longitudinal axis of the arm [13], whilst avoiding transection of a sucker. The cut face of the transected arm was immersed over a length of ~1 cm in local anaesthetic (3 % mepivacaine; Carboplyina, Curaden Healthcare SRL) for 5 min. Previous studies have shown that nerve block can be produced in *O. vulgaris* by topical application of the local anaesthetic agent xylocaine [35] and topical application of a related agent mepivacaine (3 %) blocks transmission in the axial nerve cord (unpublished observations). It should be noted that there are no systemic analgesics with demonstrated efficacy in cephalopods (reviewed in [29, 31]) and that previous studies using surgical transection of arms or tentacles in cephalopods have not provided any post-surgical analgesia or local anaesthesia [19, 22, 36]. Animals were then placed in a container with fresh sea water and monitored until ventilation and the righting reflex had recovered (15–30 min), after which animals were returned to their home tank until they were killed 2 h (*n* = 6), 6 h (*n* = 6) or 24 h (*n* = 7) from the time of transection. Animals were inspected regularly in their home tank after surgery. When the animals were returned to the home tank there was no obvious behaviour change and the latency to attack a crab was not significantly different between the three groups prior to lesion, and following lesion.

Animals were killed 2, 6, or 24 h post-lesion by prolonged (>30 min) immersion in a 3.5 % solution of magnesium chloride hexahydrate in sea water [34]. Breathing as indicated by mantle contraction normally ceased within 15 min [37]. Death was confirmed by mechanical destruction of the brain except in studies where the brain was required for subsequent analysis, in which case the dorsal aorta and systemic heart were transected. This methodology is consistent with current guidelines [31].

### Macroscopic and ultrasound assessments

Immediately following transection (0 h) and at the time of sacrifice (2, 6, 24 h) the cut face of the arm was photographed (Canon Eos 600D, Sigma 105 mm 2:2.8 DG Macro lens). The entire transected face of the arm was white in appearance due to exposure of the arm musculature with the central nerve cord visible and the skin partially retracted from the cut edge. Wound healing was assessed macroscopically by quantification of the white area visible at 6 h and 24 h. The area was measured from the high-resolution photographs using Image J software (National Institutes of Health, USA) and expressed as a percentage of the area at 0 h (100 %). Data are expressed as mean values with standard deviation (SD). A Kruskal-Wallis test (GraphPad Prism 6) was used for statistical analyses, and significance was inferred at *p* < 0.05.

For one animal, the transected tip of an arm was imaged under general anaesthesia immediately following surgery and again 24 h later in the same animal using high-resolution ultrasound (Vevo 2100 Visualsonics, The Netherlands). Ultrasound has previously been used to image the structure of the arm in *O. vulgaris* with and without general anaesthesia [37].

### Histology

For all electron microscopy (EM), tissues were fixed in 2.5 % gluteraldehyde in seawater, post-fixed in 1 % osmium tetroxide, and washed in 0.2 mol/L sodium cacodylate buffer. For scanning EM, samples were then dehydrated through a series of ascending concentrations of ethanol, critical point dried using an Emitech K850, mounted on stubs, and sputter-coated with gold (Bio-Rad E5000). Samples were viewed with a Cambridge Stereoscan 360 and images acquired with Image Access (V.3). For transmission EM, following post-fixation and washing, samples were stained en-block for 30 min in 3 % alcoholic uranyl acetate, and then dehydrated through an ascending series of ethanol. Incubation in propylene oxide for 30 min was followed by overnight infiltration in 50/50 propylene oxide resin mix (TAAB low viscosity resin). Finally, samples were infiltrated with 100 % resin (2 × 1 h) and embedded/polymerised overnight at 60 °C. Semi-thin sections (2 μm) were stained with alkaline toluidine blue (1 % dye in 1 % borax) to establish regions of interest and tissue orientation. Thin sections were cut at 90 nm (diamond knife), mounted on copper grids (uncoated 200-mesh), pretreated with 1 % aqueous potassium permanganate, stained with uranyl acetate and Sato’s lead citrate (for contrast). Viewing was achieved with an Hitachi 7100 TEM with a GATAN multi-scan digital camera. Digital Micrograph software was used for image acquisition.

For analysis by standard microscopy, tissue fixed in 4 % paraformaldehyde in sea water was processed for routine wax embedding and sectioned at 10 μm. Sections were de-waxed and rehydrated through a series of descending concentrations of ethanol and stained using haemotoxylin and eosin (H&E), periodic acid-Schiff (PAS), or for Terminal deoxynucleotidyl transferase dUTP nick end labelling (TUNEL) according to manufacturers’ instructions (Roche) to reveal dying cells.

## Results

### Macroscopic assessment of wound closure

In order to study the acute wound response in *O. vulgaris*, the distal 10 % of an arm (primarily L2) was amputated under general anaesthesia. There was no arm withdrawal in response to the transection, but the proximal arm recoiled from the transected distal tip by a few millimetres, indicating that the arm was under some longitudinal tension. The skin did not appear to retract from the wound site, nor did the central muscle or nerve cord protrude; histologically however (description below), there was evidence of skin wrinkling, again indicating that the tissue was under some tension that was lost upon transection. No overt bleeding was observed, although this is difficult to detect as cephalopod blood is virtually colourless when de-oxygenated and pale blue when oxygenated [38].

**Fig. 1:**
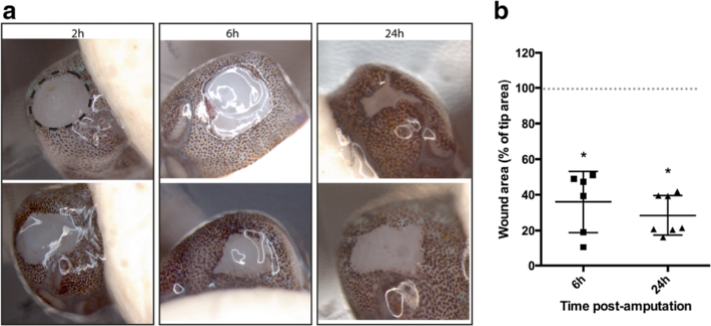
Dynamics of wound closure. **a** Images of transected stumps at 2, 6, and 24 h were acquired using a dissection microscope, and 2 biological replicates at each time-point are shown. **b** Wound area was calculated using ImageJ software, and is presented as a percentage of the area of the stump at the time of amputation (calculated using width (diameter) of an assumed “circular” arm). Calculations were performed by two independent reviewers, mean ± SD was plotted, and compared using a Kruskal-Wallis test (*p < 0.05, significant compared to 0 h wound size)

To quantify and plot the time-course of wound closure, we exploited the white appearance of the exposed arm musculature/central nerve cord of the transected face of the arm at 6 and 24 h and compared this with the area of the wound at 0 h (immediately after amputation). Qualitatively, at 2 h there was already a notable reduction in wound size (Fig. 1a). At 6 h, significant wound closure was observed (64.0 ± 17.2 % closure; mean ± standard deviation (SD), *n* = 6), but interestingly, only modest additional progress was made in the 6–24 h time-frame (71.5 ± 11.1 % closure, *n* = 7). This may be in part attributable to the inter-animal variability in rate of closure, as the data revealed two distinct populations of “faster” and “slower” healers, at both 6 and 24 h (Fig. 1b).

### Wound contraction and cell migration contribute to rapid closure

**Fig. 2:**
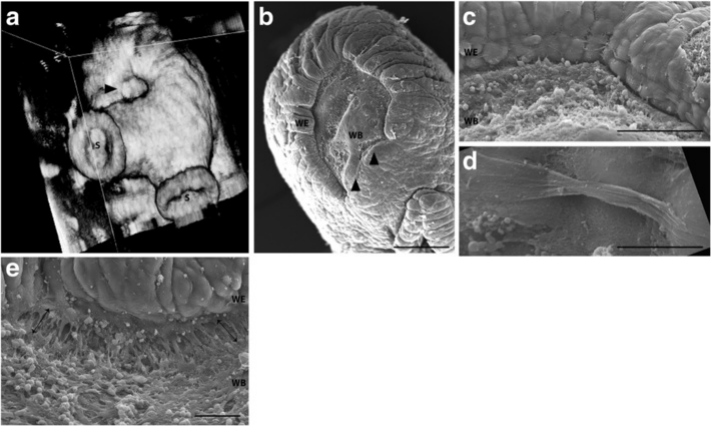
Wound closure mediated by purse-string contraction. **a**) High-resolution ultrasound imaging of a 24 h wound confirmed that the marginal skin was drawing closed over the musculature and nervous tissue constituting the core of the arm (S, suckers; arrowhead, wound). **b** Equally, scanning EM showed tissue puckering at the wound margin, which was incomplete ventrally (arrowheads). Higher magnification analysis revealed: 1) that each tissue fold consisted of two, three or more cell widths **c**; 2) that the wave-like advancing epithelial margin (wound-edge epithelium, WE) is at least two cell heights **c**; and 3) a belt-like structure that may constitute a contractile cable, could be observed between some of the cells **d**). **e** At 24 h, basal wound edge epithelial cells (WE) displayed continued adhesion to their neighbours, lamella on the leading edge, and cell adhesions to the underlying wound matrix (arrows), suggestive of collective cell migration. WB, wound bed. Scale bars: **b**, 500 μm; **c**, 100 μm; **d**, 10 μm; **e**, 50 μm

Gross analysis of the amputation wounds at 24 h revealed a puckered appearance of the skin at the wound margin (Fig. 1a), suggesting that a purse-string-like contractile mechanism may contribute to wound closure. High-resolution ultrasound imaging of a 24 h wound confirmed that the marginal skin was raised and drawing closed over the musculature and nervous tissue constituting the core of the arm (Fig. 2a). Equally, scanning electron microscopy (EM) at the same time-point showed tissue puckering at the wound margin (Fig. 2b). Higher magnification analysis revealed: 1) that each tissue fold consisted of two or more cell widths (Fig. 2c); 2) that the wave-like advancing epithelial margin is at least two cell heights (Fig. 2c); and 3) a belt-like structure that may constitute a contractile cable, could be observed between some of the cells (Fig. 2d). Notable was the incomplete contractile response around the circumference of the wound, with very little gathering observed on the ventral (sucker) side (Fig. 2b).

Again using scanning EM, analysis of the cells at the advancing wound margin at 6 h post-amputation showed cell protrusions indicative of active cell migration. The epithelial cells at the leading edge had abundant and obvious attachments to the wound bed at 24 h (Fig. 2e).

### A dense cell-rich plug provides wound coverage

**Fig. 3:**
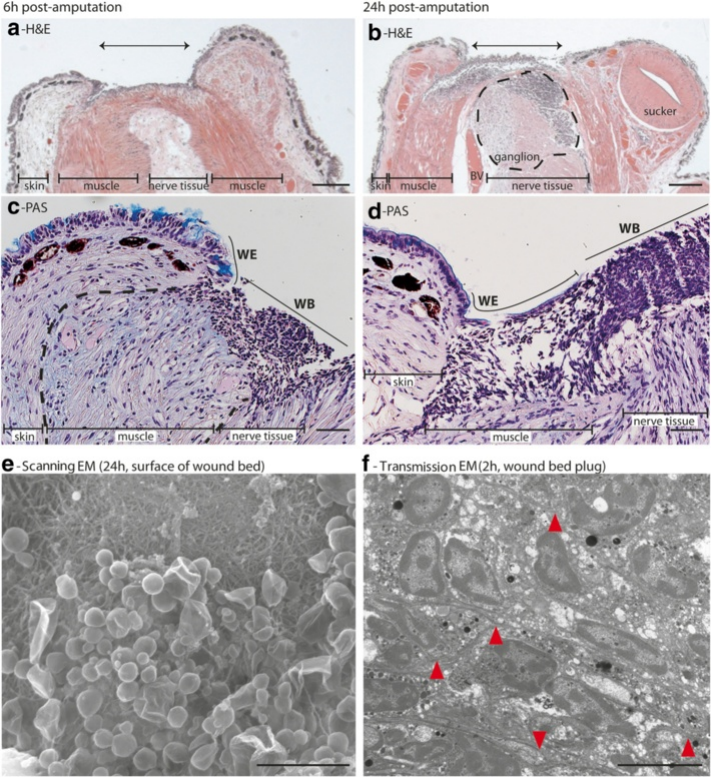
A cellular plug rapidly covers exposed tissue. (**a**, **b**) H&E and **c**, **d** Periodic Acid-Schiff staining of healing amputation wounds. An increasing cellularity of the wound bed was seen during the time-course studied. PAS confirmed that this is not simply mucous secretions. Arrow overlies central tissue and highlights width of wound. WE, wound-edge epithelium; WB, wound bed. **e** Scanning EM showed diverse sub-cellular-sized structures on the surface of the plug (e.g. extracellular vesicles, mucus). **f** Transmission EM revealed the densely packed and tightly interdigitated cells (arrowheads show finger-like processes of cell membrane) comprising the deeper plug. Scale bars: **a**&**b**, 200 μm; **c**&**d**, 50 μm; **e**, 5 μm; **f**, 10 μm

Macroscopically, we did not consistently observe protrusion of the central muscle and nervous tissue beyond the surrounding skin. At 2 h post-amputation, there was a varied degree of extension, with some showing significant exposure, and others none at all (Additional file 1: Figure S1). This appeared to affect rate of closure, with the least protrusion correlating with greatest closure in the 2 h time-frame. For all 2 h specimens, the most distal skin appeared somewhat detached from the underlying muscle layer (Additional file 1: Figure S1), an observation that was no longer obvious at 6 h (Fig. 3). Variably at 2 h, but consistently by 6 h, an abundance of cells coalesced over the exposed tip of the amputated arm, and even accumulated in the plane between the exposed muscle and the overlying skin. A distinct advancing simple epithelial cell layer over a portion of the plug (Fig. 3d) suggests a non-epithelial origin of the plug cells. Scanning EM of the wound bed at 24 h provided some insight into the composition, revealing abundant extracellular matrix and structures smaller than cells that may be extracellular vesicles and/or mucous. Certainly the bulk of the plug was comprised of cells (based on the haematoxylin-positive nuclei, and lack of PAS staining of mucus in this area). Transmission EM of the cells in this region showed extensive interdigitation, indicating the strength of this structure and its ability to fulfil a protective function.

### Occurrence of cell death in multiple tissue layers

**Fig. 4:**
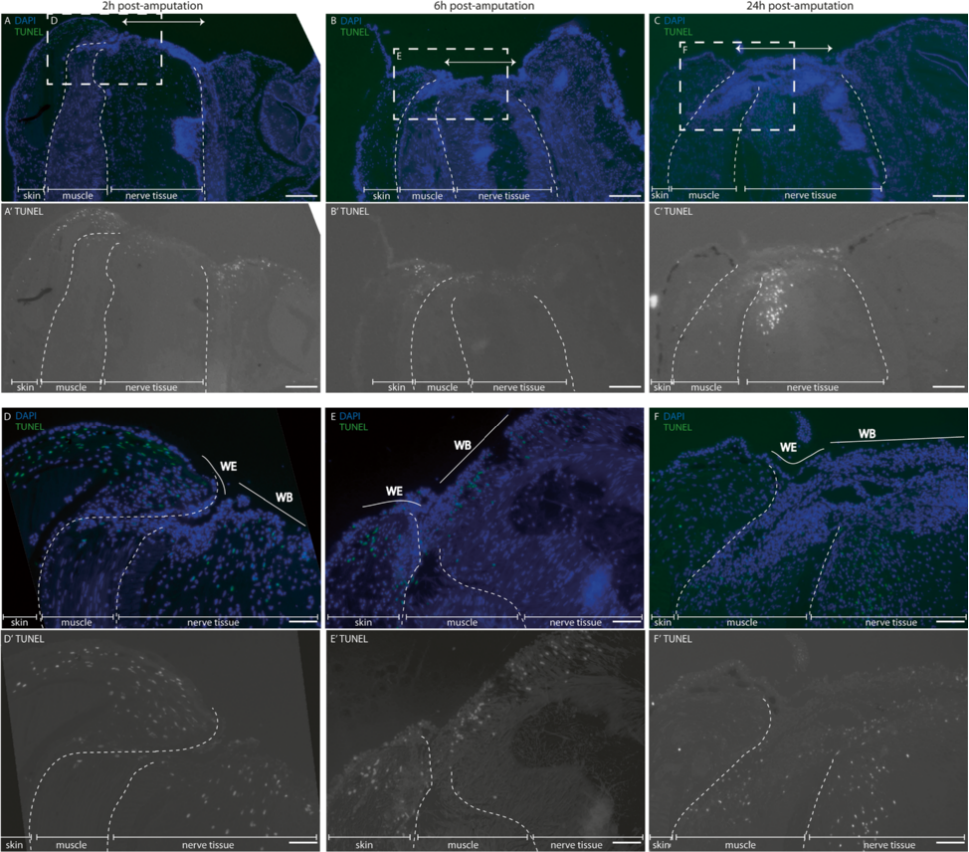
Cell death in wound-edge skin and stump. Wound margins at 2 h (**a**, **d**), 6 h (**b**, **e**), and 24 h (**c**, **f**) post amputation were stained for TUNEL (terminal deoxynucleotidyl transferase dUTP nick end labelling; green in upper panels, greyscale in lower panels), and counterstained DAPI (blue). Hatched white boxes (**a**, **b**, **c**) are magnified in (**d**, **e**, **f**). Arrow overlies central tissue and highlights width of wound. WE, wound-edge epithelium; WB, wound bed. Scale bars: **a**-**c**: 200 μm; **d**-**f**: 50 μm

As early as 2 h after amputation, there was a spread of cell death (TUNEL-positive cells) within the skin at the leading edge of the wound, across the surface of the wound bed, and a few cells apparent within the most distal portion of cut muscle (Fig. 4). At this time-point, the dying cells appeared restricted to the wound edges, and were not yet present in the tissue constituting the centre of the arm. By 6 h, cell death had spread to the centre of the wound bed, and evidence of dying cells in the cleft between the inner muscle tissue and the migrating epidermis persisted (Fig. 4). Cell death spanned the breadth of the wound bed and appeared in the central nerve cord and distal ganglion by 24 h. Although a few cells with fragmented DNA were seen in proximal musculature, it was localised to the most distal portion of the stump.

## Discussion

In Lange’s first detailed description of regeneration of cephalopods in 1920, three stages were identified: wound healing, degeneration, and renewal [13]. Since then, and with a recent resurgence in this topic, there have been a few and increasingly molecular descriptions of arm regeneration in the octopus [19] and other cephalopods [9, 21, 22]; however, the acute wound response has not been the focus of these investigations. The present study focused on the initial wound healing and degeneration events occurring in the first 24 h after removal of the distal 10 % of an arm in *O. vulgaris.* This model represents an extent of injury that would be commonly experienced in the wild [6], thus it is considered ecologically relevant [8]. Moreover, the events occurring in this time frame are functionally essential in “setting the scene” for subsequent limb regeneration [23, 24].

Immediately following injury, the arm recoiled slightly from the dissected tip, suggesting some tension in the tissue; however, there was no consistent protrusion of either the central nerve cord or muscle as previously described [9, 13]. This may be attributable to differences in anaesthesia, the magnitude of the injury, or the state of tension of the arm muscles and/or skin, which may be related to the proximity of the nearest sucker and could potentially affect subsequent repair dynamics.

Assessment of the temporal dynamics of gross wound closure over 24 h showed dramatic contraction of the skin tissue over the wound as early as 2 h, and that translated into a remarkable >60 % closure by 6 h. This was only marginally increased in the subsequent 18 h, with approximately 70 % of the tip covered after one day. The extensive closure within one day in octopus aligns well with those previously reported in other cephalopods. For example, 3–5 days [9] or 5–7 days [21] were required for complete re-epithelialisation following arm amputation in cuttlefish (*S. officinalis*), a time-course which may be influenced by species, water temperature, and animal age, state of health, size and source (e.g. wild *vs* captive bred, [9, 21]). Interestingly, our specimens appeared to segregate into two groups, with approximately equal numbers of “faster” and “slower” healers. Although the biological replicates are low in number, this appeared independent of the sex or weight of the animals, although the influence of sucker proximity and tissue tension, nor age of the animal could be assessed [27].

Ultrasound and scanning EM assessment of the wound revealed contraction as a significant mechanism underlying the rapid closure. The reaction of the skin musculature is thought to contribute to these tissue movements [39], but an epithelial component was also observed. As first described by Lange [13] and re-iterated by Feral (in the study of cuttlefish arms, [9]) and Polglase et al. (in the study of mantle repair in octopus, [20]), we saw a crest of tissue, a “raised lip”, which could protect the exposed tip. Furthermore, scanning EM allowed us to observe a belt-like structure at the wound perimeter that we suggest may be likened to a contractile actin purse-string. Characterisation of the composition of this apparatus in future studies (for example with phalloidin staining for filamentous actin) would provide insight into the mechanism of this phase of closure, and has the potential to highlight an evolutionarily conserved feature of wound repair [40]. The incomplete contractile apparatus around the circumference of the wound (lacking ventrally) was intriguing in that this difference in tension may permit a dorsal shift in the wound, pulling suckers over the end of the arm. This “abnormal positioning of the suckers” [13] is predicted to restore some level of functionality to the arm very quickly, despite the shortened length [21]. We suggest that this early asymmetric wound contraction also underpins the consistently dorsal orientation of regrowth described for amputation injuries caused experimentally [13], or which occurred in the wild [6].

Tissue contraction made a significant contribution to wound closure in the first 6 h, but then its involvement in further healing lessened. As contraction plateaued, cell protrusions suggestive of active cell migration at the epithelial margin were seen. Tissue contraction, cell migration, and cell morphology changes together are thought to be sufficient for closure of a reasonably-sized wound, in the absence of proliferation [9, 13, 19, 21]. This is perhaps analogous to mechanisms of epithelial restitution during repair of mammalian gastrointestinal mucosa [41], and also to the tissue dynamics observed during dorsal closure and wound repair in *Drosophila* embryos [42]. Although cell proliferation has not been analysed in our samples, based on cuttlefish studies, cell division is thought to become important during the regenerative phase starting around day 5 after injury [21].

Histological analysis of the wounds revealed a coalescing of cells over the exposed tip of the amputated arm. These cells were impressively interdigitated, creating a seemingly impermeable temporary protective covering. This may be equivalent to the interdigitation of platelets during the haemostatic response after vertebrate skin wounding [43]. Of course this structure is distinct from the vertebrate clot, in that its destiny is not to be sloughed off, but retained, likely contributing to the blastema [9, 13]. The identity of the cells comprising this plug has historically been assumed to be blood cells [9, 13], and specifically not muscle cells [13]. The prevailing view is that amoebocytes/haemocytes make a significant contribution [39, 44, 45]; however, our histology data (e.g. Fig. 3b) suggest that muscle cells may also play a part. Whether muscle de-differentiation contributes to regeneration appears to be highly species–specific (e.g. occurring in newt, but not axolotl [46]). Coupling information about cell proliferation in the plug with emerging molecular signatures of distinct cell populations and culture strategies should allow us to study both the origins [47] and plasticity of these cells.

The matrix composition of the plug is now widely accepted to be functionally important for regeneration in many species, from mouse ear [48] to axolotl limb [49]. Interestingly, Lange [13] almost 100 years ago may have been one of the first to hypothesize that matrix susceptibility to proteolytic degradation (or resistance, as in fibrosis) could affect regenerative capacity [50]; octopus arm may be an ideal model for the study of wound-associated extracellular matrix features permissive of regeneration.

From the first descriptions of cephalopod response to limb amputation injury, it is known that there is “degeneration” or “disintegration” of muscle and nerve tissue [9, 13, 21]. Since that time, work on other regenerating species has revealed that this is likely to be attributable to apoptotic cell death, which is essential for blastema formation and activating the proliferative response required for regeneration [51]. In this study, the mechanism of cell death was not confirmed, however, cell death based on TUNEL-positivity was seen in skin, muscle and nerve cells, with varying temporal dynamics, but all within six hours.

As recently revealed by a study of axolotl skin [52], a comparison of wound repair in a regenerating tissue (e.g. octopus arm) with a site which only displays wound healing (e.g. octopus mantle [20]) could improve our understanding of conserved cellular and molecular mechanisms, and possibly also the impact of tissue functionality in driving evolution of regeneration [8]. In both the transected arm and the wounded mantle, skin tissue infolding and contraction was a major contributor to early closure. Mantle repair may be distinct from our observations in the arm in that the cellular plug was partially excluded during re-epithelialisation, and cell death was specifically described to be necrotic [20]. These differences are thought to reflect the absence of a blastema in the mantle, and we are intrigued as to whether or not absence of chromatophore regeneration in the mantle [20] contributes to the “healing” as opposed to “regenerative” environment.

## Conclusion

Herein, we have described the acute response of *O. vulgaris* to amputation injury. Repair to an arm wound occurred rapidly, with almost complete closure occurring in some individuals in 6–24 h. The observed mechanisms of tissue repair included tissue contraction, epithelial cell migration, and a coalescing of cells at the site of injury, which are notably evolutionarily conserved features of the process. Using this model, which mimics an injury relevant to the life of the animal, we have identified key early processes of repair preparatory to regeneration. The complexity of the tissue architecture of the *O. vulgaris* arm that includes epithelial, chromatophore, vascular, muscle and neural tissue makes this a particularly interesting system in which to study acute responses to injury and subsequent regeneration.

### Ethics approval and consent to participate

The following text appears in the Methods section of the manuscript:

*At the time the studies were performed, no national legislation was in place in Italy for regulation of research involving cephalopods. The care and welfare assessment of animals was consistent with best practice at the time the study was undertaken* [29, 30] *and took into account the then impending changes in the European Union legislation bringing cephalopods within the scope of regulations covering scientific research involving living animals (Directive 2010/EU/63,* [25, 26, 31]*). Experimental design and animal care was discussed with the institution veterinarian who also monitored the study.*

### Consent for publication

Not applicable.

## Additional file

Additional file 1: Figure S1. Variable tissue reaction to amputation, without consistent protrusion. (A) Gross observations illustrate the range of response (two biological replicates). (B) Histological assessment at 2 h (H&E; three biological replicates) showed protrusion of the central muscle and nervous tissue in 1 of 3 samples (arrow overlies central tissue and highlights width of wound). Scale bars: A, 2 mm; B, 200 μm. (TIF 31650 kb)

## Abbreviations

EM: electron microscopy
H&E: haematoxylin and eosin
PAS: periodic acid-schiff
SD: standard deviation
SEM: standard error of the mean
TUNEL: terminal deoxynucleotidyl transferase dUTP nick end labelling
