# Supplementary figures and images for: Mechanisms of wound closure following acute arm injury in Octopus vulgaris
Source: Zoological Lett. 2016 Mar 29;2:8. doi: 10.1186/s40851-016-0044-5 (PMC4812652; doi:10.1186/s40851-016-0044-5)

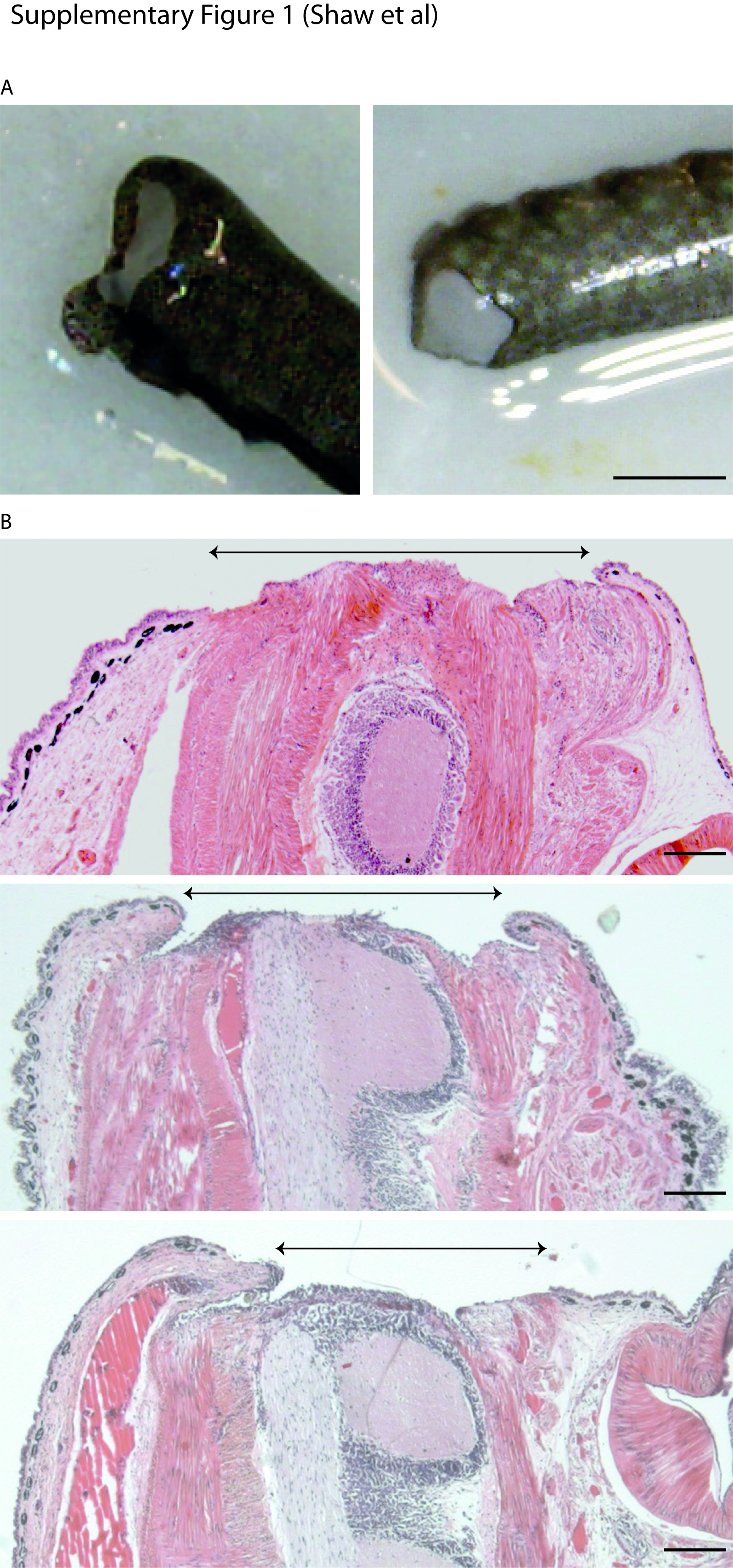

Supplement: Additional file 1: Figure S1. — Variable tissue reaction to amputation, without consistent protrusion. (A) Gross observations illustrate the range of response (two biological replicates). (B) Histological assessment at 2 h (H&E; three biological replicates) showed protrusion of the central muscle and nervous tissue in 1 of 3 samples (arrow overlies central tissue and highlights width of wound). Scale bars: A, 2 mm; B, 200 μm. (TIF 31650 kb) [file 40851_2016_44_MOESM1_ESM.tif]
